# Efficacy of therapist-delivered transdiagnostic CBT for patients with persistent physical symptoms in secondary care: a randomised controlled trial

**DOI:** 10.1017/S0033291721001793

**Published:** 2023-01

**Authors:** Trudie Chalder, Meenal Patel, Matthew Hotopf, Rona Moss-Morris, Mark Ashworth, Katie Watts, Anthony S. David, Paul McCrone, Mujtaba Husain, Toby Garrood, Kirsty James, Sabine Landau

**Affiliations:** 1 Department of Psychological Medicine, Institute of Psychiatry, Psychology and Neuroscience, King's College London, London, UK; 2 Health Psychology Section, Institute of Psychiatry, Psychology and Neuroscience, King's College, London, UK; 3 Faculty of Life Sciences and Medicine, School of Population Health and Environmental Sciences, King's College London, London, UK; 4 Division of Psychiatry, UCL Institute of Mental Health, Maple House, 149 Tottenham Court Road, London W1T 7NF, UK; 5 Institute for Lifecourse Development, Old Royal Naval College, University of Greenwich, Park Row, Greenwich, London SE10 9LS, UK; 6 UK South London and Maudsley NHS Foundation Trust, London, UK; 7 Guy's Hospital, Great Maze Pond, London SE1 9RT, UK; 8 Department of Biostatistics and Health Informatics, Institute of Psychiatry, Psychology and Neurosciences, Psychology and Neuroscience King's College, London, UK

**Keywords:** Cognitive behavioural therapy (CBT), medically unexplained symptoms, persistent physical symptoms, randomised controlled trial (RCT), secondary medical care, transdiagnostic

## Abstract

**Background:**

Medically unexplained symptoms otherwise referred to as persistent physical symptoms (PPS) are debilitating to patients. As many specific PPS syndromes share common behavioural, cognitive, and affective influences, transdiagnostic treatments might be effective for this patient group. We evaluated the clinical efficacy and cost-effectiveness of a therapist-delivered, transdiagnostic cognitive behavioural intervention (TDT-CBT) plus (+) standard medical care (SMC) *v.* SMC alone for the treatment of patients with PPS in secondary medical care.

**Methods:**

A two-arm randomised controlled trial, with measurements taken at baseline and at 9, 20, 40- and 52-weeks post randomisation. The primary outcome measure was the Work and Social Adjustment Scale (WSAS) at 52 weeks. Secondary outcomes included mood (PHQ-9 and GAD-7), symptom severity (PHQ-15), global measure of change (CGI), and the Persistent Physical Symptoms Questionnaire (PPSQ).

**Results:**

We randomised 324 patients and 74% were followed up at 52 weeks. The difference between groups was not statistically significant for the primary outcome (WSAS at 52 weeks: estimated difference −1.48 points, 95% confidence interval from −3.44 to 0.48, *p* = 0.139). However, the results indicated that some secondary outcomes had a treatment effect in favour of TDT-CBT + SMC with three outcomes showing a statistically significant difference between groups. These were WSAS at 20 weeks (*p* = 0.016) at the end of treatment and the PHQ-15 (*p* = 0.013) and CGI at 52 weeks (*p* = 0.011).

**Conclusion:**

We have preliminary evidence that TDT-CBT + SMC may be helpful for people with a range of PPS. However, further study is required to maximise or maintain effects seen at end of treatment.

## Introduction

Medically unexplained symptoms (MUS) otherwise referred to as persistent physical symptoms (PPS) are symptoms with no clear-cut organic cause (Deary, Chalder, & Sharpe, 2007; Edwards, Stern, Clarke, Ivbijaro, & Kasney, 2010). They are associated with psychological distress, functional impairment, and high health costs (Bermingham, Cohen, Hague, & Parsonage, 2010; Nimnuan, Hotopf, & Wessely, 2001a; Poloni et al., 2018). We chose to use the term PPS over MUS primarily as patients and the public preferred it over other labels (Marks & Hunter, 2015; Picariello, Ali, Moss-Morris, & Chalder, 2015). The term functional somatic disorder (Burton, Fink, Henningsen, Löwe, & Rief, 2020), has been suggested by experts, as there are a range of underlying pathophysiological changes, evident in these groups. Although we agree with this assertion the term functional is not understandable to all and was not the patients' choice.

The prevalence of PPS is common in both primary and secondary care (Poloni et al., 2018). It is estimated that almost half of patients in primary care report at least one PPS (Haller, Cramer, Lauche, & Dobos, 2015) and PPS accounts for approximately 52% of new referrals in secondary care (Nimnuan, Rabe-Hesketh, Wessely, & Hotopf, 2001b). Although patients are likely to have had confirmation that their symptoms are medically unexplained by medical specialists (Burton, McGorm, Weller, & Sharpe, 2011b; McGorm, Burton, Weller, Murray, & Sharpe, 2010; Reid, Wessely, Crayford, & Hotopf, 2002), approximately 1% of patients with PPS (adults aged <65) are repeatedly referred from primary to secondary care (Burton, McGorm, Weller, & Sharpe, 2011a; Burton et al., 2011b).

PPS encompasses a range of symptoms/syndromes including fibromyalgia, irritable bowel syndrome (IBS), chronic fatigue syndrome (CFS), dizziness, non-cardiac chest pain and tension headaches (Henningsen, Zipfel, & Herzog, 2007). They are seen in all secondary care settings including rheumatology, gastroenterology, cardiology, respiratory and neurology (Chalder & Willis, 2017). The severity of symptoms can vary widely from relatively mild symptoms to multiple/chronic debilitating symptoms (Gerger, Hlavica, Gaab, Munder, & Barth, 2015). Patients with PPS can develop unhelpful cognitions and behaviour which can consequently lead to a reduction in daily functioning, reduced quality of life, and an increased susceptibility towards developing depression and anxiety (Chalder & Willis, 2017).

There have been a number of systematic reviews/meta-analyses published that assess the evidence for cognitive behavioural therapy (CBT) for PPS (Jones & de C. Williams, 2019; Kleinstäuber, Witthöft, & Hiller, 2011; Liu, Gill, Teodorczuk, Li, & Sun, 2019; Menon, Rajan, Kuppili, & Sarkar, 2017; Sumathipala, 2007; Van Dessel et al., 2014). They all concluded that CBT interventions were effective in terms of improving outcomes when compared to control conditions. The most recent, a meta-analysis published by Liu et al. (2019) assessed the efficacy of CBT for somatoform disorders and PPS which are assumed to be synonymous, and concluded that CBT was superior to usual care, enhanced care or waiting list in reducing somatic, depressive, and anxiety symptoms as well as improving physical functioning (Liu et al., 2019). Fifteen randomised controlled trials (RCTs) were included which were heterogeneous in terms of trial design, patient populations, intervention characteristics, outcome measures, therapist characteristics, and follow-up periods. Factors that possibly facilitated the effectiveness of CBT were manualised treatment and each session lasting more than 50 min. However, effect sizes varied depending on the outcome in question in each study. For example, two separately conducted reviews by Van Dessel et al. (2014) and Kleinstäuber et al. (2011) reported smaller effect sizes for symptom severity than functional impairment, when comparing CBT to usual care or waiting list (Kleinstäuber et al., 2011; Van Dessel et al., 2014).

There is evidence to suggest that there is considerable overlap between PPS syndromes. A review by Aaron and Buchwald (2001) emphasised the substantial overlap between 12 unexplained syndromes which included patients with CFS, fibromyalgia and IBS (Aaron & Buchwald, 2001). Many patients who had IBS were also diagnosed with fibromyalgia (32–80%), CFS (58–92%) and temporomandibular pain (64%). More recently, Petersen et al. (2020), in their population study, found functional somatic syndromes to be highly prevalent and overlapping with multi-syndromic cases being most affected (Petersen et al., 2020). Also for overlapping symptoms there are a range of common psychological and behavioural responses associated with PPS including ways of thinking, feeling, and habitually responding (Chalder & Willis, 2017; Olde Hartman et al., 2018). Transdiagnostic theory suggests that by targeting these common processes the same treatment can be used across different symptom clusters with flexibility to address symptom specific issues (Mansell, Harvey, Watkins, & Shafran, 2009). Interventions that target transdiagnostic processes have the potential to use less resource than targeted interventions. Furthermore, a transdiagnostic approach may be more appropriate and acceptable in a clinical setting where patients often have heterogeneous problems (Norton & Roberge, 2017).

We designed a trial Persistent physical symptoms Reduction INtervention: a system Change and Evaluation in secondary care (PRINCE Secondary) to assess the efficacy and cost-effectiveness of a therapist-delivered, transdiagnostic CBT (TDT-CBT) intervention plus (+) standard medical care (SMC) *v.* SMC alone for the treatment of patients with PPS in secondary medical care.

## Methods

### Study design and participants

This was a two-arm RCT, with assessments carried out at baseline and at 9, 20, 40- and 52-weeks post randomisation for patients with PPS. Patients were recruited from secondary care clinics in the UK National Health Service between August 2015 and January 2018. PPS were diagnosed by an experienced consultant physician after a comprehensive assessment and medical history had been undertaken and investigations carried out. All patients received routine and specialist investigations in the specialist secondary care clinic before a diagnosis was made. PPS were diagnosed by clinicians working in rheumatology, cardiology, respiratory, neurology and gastroenterology clinics. If there was any doubt about the patient's diagnosis it was discussed by a physician and psychiatrist. Operational criteria for each functional problem was not used as it was deemed to be impractical and of limited benefit due to the diverse nature of the problems included. Recruitment into the study was undertaken by the research team using an eligibility checklist (see inclusion criteria) and if eligible, the study was discussed with the patient and those who agreed were asked to complete a consent form. Patients who provided consent and baseline data within 1 month of screening were randomised to either TDT-CBT + SMC or SMC alone. For those recruited into the trial outcome data collection was completed in January 2019. The trial registration can be found at ClinicalTrials.gov (NCT02426788) and the protocol has been published (Chalder et al., 2019).

Inclusion criteria were: (i) patients aged between 18 and 65 years [age increased to 70 on advice of trial steering committee (TSC) once trial had commenced recruitment] with a PPS (e.g. fibromyalgia, IBS, persistent cough, and non-cardiac chest pain); (ii) a score ⩾10 on the Work and Social Adjustment Scale (WSAS), (iii) ability to read and write in English, (iv) willingness to complete all trial visits and (v) willingness to give written informed consent and provide baseline data. Patients were excluded if they had active psychosis and/or factitious disorder; headaches as their main and only PPS symptom (given the clinical complexity of differentiating headaches and migraine, headaches were excluded); non-epileptic seizures as their main and only PPS symptom [due to an ongoing RCT at the time of recruitment that was evaluating a specific cognitive behavioural approach for Dissociative Seizures, now published (Goldstein et al., 2020)]; primary drug or alcohol dependence disorder; benzodiazepine use exceeding the equivalent of 10 mg diazepam per day; received or were receiving CBT (based) interventions for PPS during the past year; at imminent risk of self-harm or participated in PRINCE Primary study (Trial Registration Number: NCT02444520). Ethical approval was obtained from the Camberwell St Giles Research Ethics Committee (REC15/LO/0058).

### Randomisation and masking

Participants were randomly allocated to either TDT-CBT + SMC or SMC with an allocation ratio of 1:1 using randomly varying block sizes, stratified by clinic (e.g. cardiology, neurology, etc.) and disability level [moderately severe impairment (⩽20) or significant impairment (>21)] as indicated by the WSAS at the time of screening. Patient randomisation was conducted following participant consent and provision of baseline data using an online randomisation system provided by the King's College London Clinical Trials Unit, UK. The system informed the trial manager of the randomisation outcome who then informed the participant and therapist of the outcome via telephone/email.

The trial manager, participants and therapists were not blind to treatment allocation. Researchers were masked. The senior statistician and chief investigator were partially blind (i.e. only knew the two groups as A and B) until the final stages of the analysis. The trial statistician was unblind.

### Description of intervention and standard medical care

Participants randomised to TDT-CBT + SMC received eight, 1-h CBT sessions over a period of 22 weeks. In addition, they also received a detailed self-help manual including homework tasks. Sessions were delivered by a qualified trial therapist. The sessions were structured and addressed four different areas: (1) engagement and rationale giving; (2) reducing avoidance by exposure techniques; (3) dealing with symptom-related cognitions and emotions; and (4) relapse prevention. Both groups received SMC which was a continuation of any planned follow-up consultations with specialised health care professionals. Participants randomised to SMC were sent the patient manual (no therapy support) at the end of their 52-week follow-up.

### Outcomes

The primary and secondary trial outcomes are summarised below. Further details on all assessments including process variables are described in the published protocol (Chalder et al., 2019). Participants were given the option to complete assessments over the telephone, in person or via post. Questionnaires were administered at baseline (pre-randomisation) and at 9, 20, 40- and 52 weeks post randomisation unless stated otherwise.

### Primary outcome

*The Work and Social Adjustment Scale* (*WSAS*) at 52 weeks measured the impact of PPS on work, home management, social leisure and private leisure activities, family, and other relationships (Mundt, Marks, Shear, & Greist, 2002). Items were rated on an 8-item Likert scale (0 (not affected) to 8 (severely affected)) with a total possible score of 40 where a higher score indicated severe impairment. This was chosen as the primary outcome because the focus of therapy was on targeting processes which might result in a reduction of the impact of symptoms.

### Secondary outcomes

WSAS measured at 9, 20- and 40 weeks post randomisation were secondary outcomes.

*The Patient Health Questionnaire 15* (*PHQ-15*) at 52 weeks measured symptom severity (Kroenke, Spitzer, & Williams, 2002). Items were rated on a 3-point Likert scale (0 = not bothered at all; 1 = bothered a little; 2 = bothered a lot) with a total possible score of 30.

*The Patient Health Questionnaire 9* (*PHQ-9*) at 52 weeks measured the severity of depression (Kroenke, Spitzer, & Williams, 2001). Items were rated on a 4-point Likert scale (0 = not at all; 1 = several days; 2 = more than half the days; 3 = nearly every day) with a total possible score of 27.

*The Generalised Anxiety Disorder-7* (*GAD-7*) at 52 weeks measured the severity of anxiety (Spitzer, Kroenke, Williams, & Löwe, 2006). Items were rated on a 4-point Likert scale (0 = not at all; 1 = several days; 2 = more than half the days; 3 = nearly every day) with a total possible score of 21.

*The Persistent Physical Symptom Questionnaire* (*PPSQ*) at 52 weeks was adapted from the Chest Pain questionnaire (Marks, Chambers, Russell, Bryan, & Hunter, 2014). An overall interference score was calculated using the average scores from three scales. These were (i) severity, (ii) distress and (iii) the problematic nature of the patients’ main presenting symptom (e.g. chest pain). Items were scored on a 10-point scale (from 1 = not at all to 10 = extremely).

*The Clinical Global Impression CGI-patient* at 52 weeks measured self-rated global change (Guy, 1976). A single item rated change on a 9-point Likert scale where 1 is completely recovered and 9 is could not get any worse.

In all cases, a higher score indicated greater severity.

Health service use and health-related quality of life was measured using the Client Service Receipt Inventory (CSRI) and EQ5D respectively. The results will be reported in a separate paper.

### Process variables

#### Therapy procedures

Situated at South London and Maudsley NHS Trust, three trained therapists in CBT provided all CBT sessions. Training was provided on the delivery of the intervention and group supervision was provided every month with one of the study team (TC). Therapists recorded the number of sessions, whether they were face to face or over the telephone as well as duration of each session. At the end of therapy, the therapist rated how well the participant adhered to therapy and rated the participant's adherence to homework tasks. Sessions were audio recorded for supervision as well as for treatment fidelity purposes. A sample of sessions 3 and 5 were analysed for treatment fidelity purposes. They were rated by two independent clinicians using a measure specifically adapted for this trial. All items were rated on a 7-point Likert scale. Therapeutic alliance (1 item; 1 = very poor to 7 = excellent), CBT skills (4 items; 1 = not at all to 7 = extensively and 1 item 1 = did not to 7 extensive attempts to develop homework) and overall therapist adherence to the manual (1 item; 1 = not at all to 7 = extensively) were assessed (Chalder et al., 2019).

Three additional process measures were completed and will be reported in a separate paper. These were the PSYCHLOPS, a patient-generated outcome measure (Ashworth, Evans, & Clement, 2009), the cognitive behavioural responses questionnaire (CBRQ) (Knoop, van Kessel, & Moss-Morris, 2012; Moss-Morris & Chalder, 2003; Ryan, Vitoratou, Goldsmith, & Chalder, 2018) and acceptance scale (McCracken, Vowles, & Eccleston, 2004).

#### Sample size

As per the published protocol (Chalder et al., 2019), we calculated our sample size based on a minimum clinically important difference of **−**3.6 points on WSAS comparing TDT-CBT plus SMC to SMC alone at 52 weeks (White et al., 2011). The within-group s.d. at 52 weeks was estimated to be 9.4 points giving a standardised effect size of Cohen's *d* = −3.6/9.4 = 0.38. The sample size calculation (Stata command sampsi) suggests that 161 patients per arm (322 in total) were needed to detect this effect size with 90.14% power after inflation to allow for an attrition rate of 25% and deflation for including baseline measures in the analysis model (factor 0.84 assuming a correlation between baseline and 52-week WSAS of 0.4) (Borm, Fransen, & Lemmens, 2007).

### Statistical analysis

All analyses were performed in line with the published protocol (Chalder et al., 2019). A detailed statistical analysis plan was developed by the trial team and approved by the trial steering committee before database lock. Treatment effects were estimated for each outcome measure by comparing the TDT-CBT + SMC and SMC groups in the intention-to-treat (ITT) population. As noted in the protocol, a post-randomisation measure of non-compliance with CBT (binary compliance variable: complied = participated in more than three therapy sessions) was assessed as a predictor of missing primary outcome in the intervention arm and found to be predictive (χ^2^_(1)_ = 47.7, *p* < 0.001). Therefore, multiple imputation (MI) by chained equations (MICE) was used in order to produce inferences valid under a missing at random assumption that allowed observed non-compliance to drive missingness. Univariable logistic regression was used to detect if baseline variables presented in Table 1 were predictive of missing primary outcome data, and thus should also be included in the imputation step of the MICE procedure. Only criteria one on the fibromyalgia assessment titled ‘Pain in the left side of the body, pain in the right side of the body, pain above the waist, and pain below the waist’ was associated with missingness of the primary outcome at a liberal 10% test level.

**Table 1.** Demographic characteristics of study sample

Baseline demographics		SMC *N* = 163	TDT-CBT plus SMC *N* = 161	Overall *N* = 324
Age mean (s.d.)		42.5 (12.9)	43.7 (12.3)	43.1 (12.6)
Gender, *n* (%)	Female	133 (81.6)	136 (84.5)	269 (83.0)
Ethnic background, *n* (%)	White	117 (71.8)	117 (72.7)	234 (72.2)
	Black, Asian, and minority ethnic	35 (21.5)	33 (20.5)	68 (21.0)
	Other/missing	11 (6.7)	11 (6.8)	22 (6.8)
First language, *n* (%)	English	140 (85.9)	134 (83.2)	274 (84.6)
	Other	23 (14.1)	27 (16.8)	50 (15.4)
Marital status, *n* (%)	Single	71 (43.6)	65 (40.4)	136 (42.0)
	Married/living together	71 (43.6)	70 (43.5)	141 (43.5)
	Other	21 (12.9)	26 (16.1)	47 (14.5)
Currently live with, *n* (%)	Partner/children/parents	104 (63.8)	109 (67.7)	213 (65.7)
	Alone	36 (22.1)	34 (21.1)	70 (21.6)
	Other	23 (14.1)	18 (11.2)	41 (12.7)
Have children, *n* (%)	Yes	83 (50.9)	96 (59.6)	179 (55.2)
Have elderly relatives, *n* (%)	Yes	19 (11.7)	17 (10.6)	36 (11.1)
Place of residence, *n* (%)	Owner occupied	61 (37.4)	59 (36.6)	120 (37.0)
	Renting (authority/private)	91 (55.8)	94 (58.4)	185 (57.1)
	Other i.e. flat share	11 (6.7)	8 (5.0)	19 (5.9)
Educational level, *n* (%)	None	15 (9.2)	13 (8.1)	28 (8.6)
	GCSE or equivalent	42 (25.8)	31 (19.3)	73 (22.5)
	A level or equivalent	31 (19.0)	27 (16.8)	58 (17.9)
	Degree	45 (27.6)	53 (32.9)	98 (30.2)
	Postgraduate	18 (11.0)	25 (15.5)	43 (13.3)
	Other	12 (7.4)	12 (7.5)	24 (7.4)
Member of self-help group or national patient organisation, *n* (%)	Yes	9 (5.5)	5 (3.1)	14 (4.3)
Had CBT, *n* (%)	Yes	45 (27.6)	47 (29.2)	92 (28.4)
Had physiotherapy, *n* (%)	Yes	108 (66.3)	119 (73.9)	227 (70.1)
Had other therapy, *n* (%)	Yes	69 (42.3)	84 (52.2)	153 (47.2)
Clinic, *n* (%)	Neurology	17 (10.4)	16 (9.9)	33 (10.2)
	Cardiology	12 (7.4)	11 (6.8)	23 (7.1)
	Rheumatology	80 (49.1)	79 (49.1)	159 (49.1)
	Gastroenterology	36 (22.1)	36 (22.4)	72 (22.2)
	Respiratory	15 (9.2)	15 (9.3)	30 (9.3)
	Pain	3 (1.8)	3 (1.9)	6 (1.9)
	OH	0 (0.0)	1 (0.6)	1 (0.3)
Meets all criteria for fibromyalgia assessment^a^	*N*	163	159	322
*n* (%)	Met	88 (54)	86 (54.1)	174 (54)

a Participants were instructed to complete three items relating to widespread pain. These items were categorical (Met/Not met). If all three items were met, fibromyalgia criteria were considered ‘met’.

Separate analyses were carried out for each continuous trial outcome. All MI analysis models were regressions. They included the outcome variable as the dependent variable and trial arm, stratification variables (clinic and disability level), therapist (three levels) and baseline values of the outcome as explanatory variables. MI models included all variables of the analysis model, all measures of the outcome at other time-points and predictors of missingness of the primary outcome (adherence to the intervention and criteria one from the fibromyalgia assessment). Imputations for continuous variables were generated by predictive mean matching with 10 nearest neighbours to ensure that all imputed outcome values were within the range of the observed data. One hundred imputations were used throughout.

Standardised effect sizes were computed for the effect of treatment on outcome for both primary and secondary outcomes. This was done by dividing the estimated trial arm difference by the baseline standard deviation of the measure.

A complier average causal effect (CACE) analysis was carried out for the primary outcome in an attempt to assess treatment efficacy by estimating the effect of actually receiving the intervention. CACE was estimated using instrumental variables regression with treatment offer as an instrument for treatment receipt. In this context treatment offer can be considered an instrument for the exposure treatment receipt if it affects the primary outcome only through its effect on treatment receipt.

A complete case analysis for the primary and secondary outcomes was also carried out as a sensitivity analysis, results from these analyses are provided in online Supplementary Table A.

### Patient and public involvement

Patient and public involvement representatives participated in all phases of the study design, including discussion to ensure that the trial was not burdensome for participants. We included an item in the clinical global change score enquiring about whether participants felt they had recovered as a result of their involvement. Early iterations of the manual were commented on specifically and modifications to language were made.

## Results

**Fig. 1.**
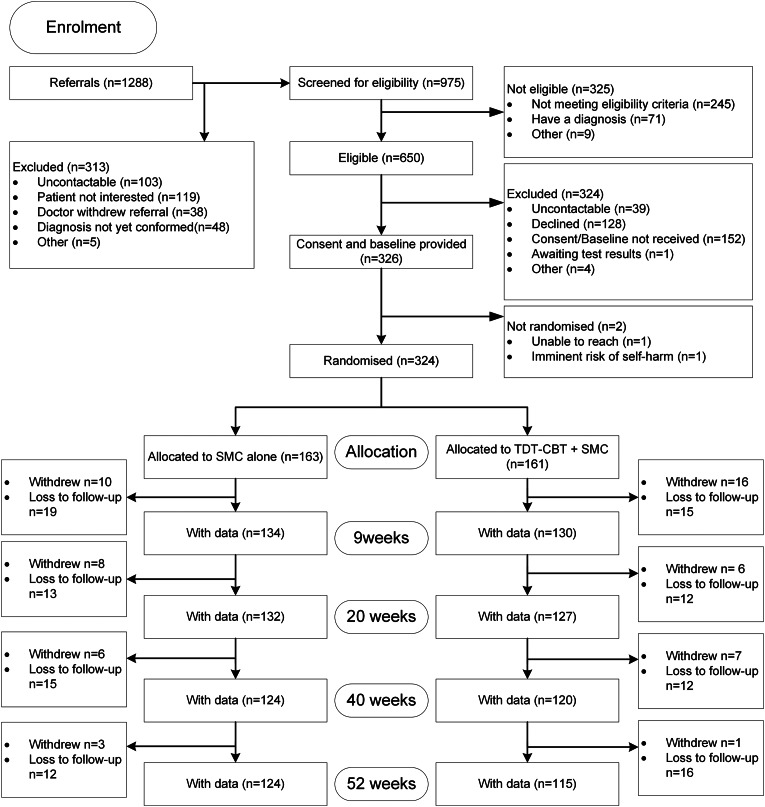
Consolidated Standards of Reporting Trials (CONSORT) diagram for Persistent Physical Symptoms Reduction Intervention in Secondary Care (PRINCE) Trial. Withdrawals refer to participants who withdrew from the trial and all further data collection.

Three hundred and twenty-four (33%) of 975 patients screened for eligibility were randomised to participate in the trial. In total, 161 participants were randomised to TDT-CBT + SMC and 163 to SMC. The Consolidated Standards of Reporting Trials (CONSORT) diagram (Fig. 1) describes the participants' journey through the study. Follow-up rates were 81% (264/324) at 9 weeks, 80% (259/324) at 20 weeks, 75% (244/324) at 40 weeks and 74% (239/324) at 52 weeks. One year follow-up was completed in January 2019.

### Baseline characteristics

Table 1 shows participants' demographic characteristic. In total, 83% were female and the mean age was 43.1 years (s.d. = 12.6). This group was predominately white and 72% had reported no previous receipt of CBT. The largest proportion of participants was recruited from rheumatology (49%). Participants reported having significant functional impairment, high levels of symptom severity and moderate levels of anxiety. In total, 72% scored above the clinical cut-off for moderate depression (PHQ-9 > 9). All baseline characteristics were well balanced between groups. A detailed breakdown of participants' clinical baseline characteristics is provided in online Supplementary Table B.

### Primary outcome measure

**Fig. 2.**
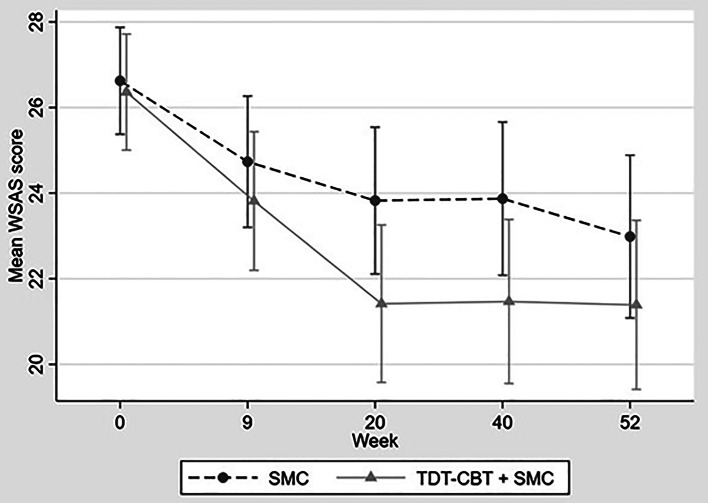
Mean WSAS score by trial arm over WSAS; range [0–40] higher score indicating more severe impairment. SMC, standard medical care; TDT-CBT, therapist-delivered, transdiagnostic cognitive behavioural therapy; WSAS, Work and Social Adjustment Scale. The figure displays mean scores of WSAS at each timepoint in both arms, note that the *y*-axis starts at 20 to aid readability.

Figure 2 shows the observed mean WSAS scores over time and by trial arm. Table 2 shows that on average across the three therapists WSAS at 52 weeks was estimated to be 1.48 points lower in the TDT-CBT + SMC arm compared to SMC [95% confidence interval (CI) for difference (−3.44 to 0.48)]; however, the effect of the intervention was neither statistically (*p* = 0.139) nor clinically significant (less than 3.6 in absolute value). The CACE estimate (a reduction of 1.63 points) was slightly larger in size than the ITT estimate but remained non-significant (*p* = 0.182).

**Table 2.** Formal trial arm comparisons for primary and secondary trial outcomes

	SMC mean (s.d.)	TDT-CBT plus SMC mean (s.d.)	Estimated treatment effect	95% CI	*p* value	Standardised difference
Primary outcome						
WSAS 52 weeks	23.0 (10.8)	21.4 (10.8)	−1.48	−3.44 to 0.48	0.139	−0.18
Secondary outcomes						
WSAS 9 weeks	24.7 (9.0)	23.8 (9.4)	−0.19	−1.56 to 1.18	0.787	−0.02
WSAS 20 weeks	23.8 (10.0)	21.4 (10.6)	−2.41	−4.36 to −0.46	0.016	−0.29
WSAS 40 weeks	23.9 (10.1)	21.5 (10.7)	−1.32	−3.19 to 0.56	0.167	−0.16
PHQ-15	15.2 (6.5)	14.0 (6.0)	−1.51	−2.70 to −0.31	0.013	−0.28
PHQ-9	12.8 (7.2)	11.0 (6.8)	−1.15	−2.64 to 0.34	0.129	−0.18
GAD-7	10.2 (6.6)	8.4 (6.3)	−1.29	−2.60 to 0.02	0.053	−0.21
PPSQ (overall score)	7.0 (2.0)	6.7 (2.0)	−0.35	−0.80 to 0.10	0.131	−0.18
CGI	5.5 (1.7)	4.8 (1.7)	−0.55	−0.97 to −0.13	0.011	−0.42

TDT-CBT, therapist-delivered, transdiagnostic cognitive behavioural therapy; SMC, standard medical care; WSAS, Work and Social Adjustment Scale; PHQ-15, Patient Health Questionnaire 15; PHQ-9, Patient Health Questionnaire 9; GAD-7, Generalised Anxiety Disorder; PPSQ, Persistent Physical Symptoms Questionnaire; CGI, Clinical Global Impression; CI, Confidence Intervals.

All inferences obtained from MICE with 100 imputations per model.

Standardised differences were calculated by dividing the estimated treatment effect by the baseline standard deviation of each corresponding outcome.

### Secondary outcome measures

**Fig. 3.**
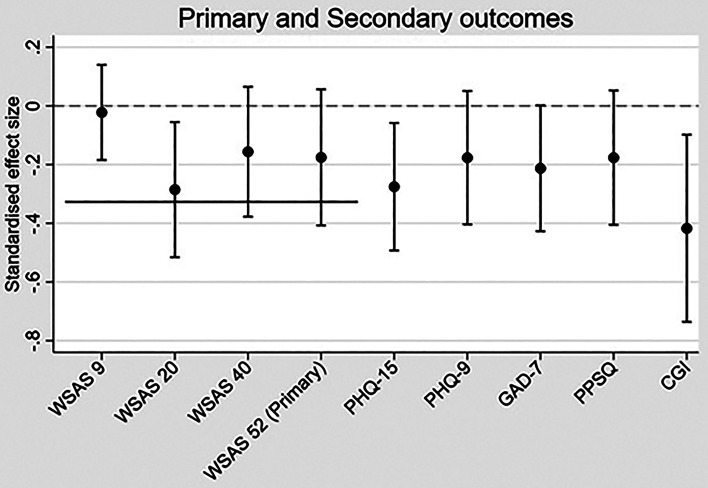
Standardised effect sizes of the primary and secondary outcomes. The solid horizontal line indicates the pre-defined minimum clinically important difference of 3.6 points on the WSAS (standardised) that was used in the sample size calculation. SMC, standard medical care; TDT-CBT, therapist-delivered, transdiagnostic cognitive behavioural therapy; WSAS, Work and Social Adjustment Scale, PHQ-15, Patient Health Questionnaire 15; PHQ 9, Patient Health Questionnaire 9; GAD-7, Generalised Anxiety Disorder; PPSQ, Persistent Physical Symptom Questionnaire; CGI, Clinical Global Impression.

Table 2 presents the results of the formal comparison of the primary and secondary trial outcomes. Mean scores of the primary and secondary outcome assessments are provided in online Supplementary Table B. Figure 3 shows the standardised effect sizes of the primary and secondary outcomes. There was a statistically significant reduction on the WSAS at 20 weeks in the TDT-CBT + SMC arm compared to SMC. The PHQ-15 at 52 weeks was also significantly lower in the TDT-CBT + SMC arm at the 5% test level. Finally, CGI at 52 weeks was found to be reduced in the intervention arm compared to the control arm. No statistically significant trial arm differences were found for WSAS at 9 and 40 weeks, PHQ-9, GAD-7 or PPSQ (see online Supplementary Fig. A for graphs showing observed mean scores over time by trial arm).

### Process variables

#### Therapy process indicators (TDT CBT + SMC only)

*Treatment attendance*: Participants attended on average 6.7 sessions out of 8 (s.d. = 2.6). In total, 135 (83.9%) were deemed compliant to the intervention (i.e. attending more than three sessions). In total, 1082 sessions were attended, and the mean length of each session was 59.2 min. Of those sessions, 68% and 32% were completed in person or via telephone, respectively. In total, 290 sessions were cancelled with sickness being the main reason (44%) and 85 sessions were recorded as ‘did not attend’ (DNA).

*Treatment adherence*: Treatment adherence scores suggest that therapists reported on average that patients adhered ‘moderately well’ to (i) treatment (mean score 2.4; s.d. = 1.2) and (ii) accepting the therapy model (mean score 2.6; s.d. = 1.1).

*Competence rating*: Mean fidelity ratings were good. Mean therapeutic alliance score was 90 (*N* = 78; range = 43–100). Mean CBT skills score was 71.9 (*N* = 78; range = 40–97). Mean therapy adherence score was 76.2 (*N* = 78; range = 14–100). These scores respectively suggest on average that the raters reported that therapists were ‘very good’ at providing support and encouragement, they often used CBT skills during the session and they often delivered therapy as described in the manual.

*Satisfaction with treatment*: Mean patient satisfaction score was 5.6 (*N* = 100, s.d. = 1.7). In total, 5% were very dissatisfied, 2% were moderately dissatisfied, 7% were slightly dissatisfied, 9% reported neither, 8% were slightly satisfied, 31% were moderately satisfied and 38% were very satisfied.

*CGI therapists*: Mean CGI therapist score was 3.7 (*N* = 140, s.d. = 1.2) suggesting that the therapist reported that their patients were between much better and a little better after therapy. Four percent were reported as recovered, 13% very much better, 20% much better, 41% a little better, 19% no change and 3% a little worse/much worse/very much worse.

#### Adverse events

There were 35 serious adverse events reported in the TDT-CBT plus SMC arm and 39 reported in the SMC arm. The spread across body system codes (i.e. the adverse event was related to the cardiovascular, endocrine, psychiatric, neurological systems, etc.) was similar between groups. There were 324 adverse events recorded in the intervention arm and 348 in the control arm with majority of adverse events being gastro-intestinal (52 in TDT-CBT + SMC, 42 in SMC), musculo-skeletal (33 in TDT-CBT + SMC, 49 in SMC), psychiatric (34 in TDT-SMC+, 46 in SMC) and immunological (48 in TDT-CBT+, 40 in SMC). An adverse event was defined as any medical occurrence, this included symptoms such as swollen eye, hip pain, flu symptoms, etc. There were 57 withdrawals from the trial, 30 from the TDT-CBT plus SMC arm and 27 from the SMC arm with the majority being due to the participant no longer wanting to take part in the trial (26 from TDT-CBT plus SMC and 26 from SMC). No adverse events were reported as being related to the intervention received.

## Discussion

This study aimed to investigate the efficacy of a TDT-CBT intervention for people with PPS in secondary care. The difference between groups in scores on the primary outcome (WSAS at 52 weeks) was not statistically significant and its size not in the clinically relevant range. Despite this, the results indicated that the estimated difference in all primary and secondary outcomes was in favour of TDT-CBT plus SMC compared to SMC alone with three out of eight secondary outcomes showing a statistically significant difference at the 5% level. These were WSAS after the end of treatment (20 weeks) and the PHQ-15 and CGI at long-term follow-up (52 weeks).

Throughout this paper, we present unadjusted *p* values. Methods for adjusting the family-wise error by methods such as the Bonferroni correction are known to be conservative. However, if one were to use a method that controlled the false-discovery rate such as the Benjamini–Hochberg procedure then the differences on PHQ-15, WSAS at 20 weeks and CGI remained statistically significant and would therefore be considered as discoveries after correction for all nine outcomes (eight secondary plus primary outcome).

A Cochrane review (Van Dessel et al., 2014) examining the effects of non-pharmacological interventions for somatoform disorders found a small and non-significant difference in functional disability and quality of life when comparing CBT to usual care at end of treatment (standardised mean difference 0.15; 95% CI −0.06 to 0.37; 4 studies, 341 participants; *I*^2^ = 0%). When other forms of psychological therapies were included (*N* = 7), an effect post treatment was found but was not sustained long term (Van Dessel et al., 2014). PRINCE Secondary found similar results such that the intervention influenced functioning at the end of therapy with an estimated effect size of 0.29 but this was not sustained over the longer-term follow-up when patients were not receiving support. In relation to symptom severity our findings replicated previous research (Liu et al., 2019). Our study showed a sustained reduction in symptom severity, measured by the PHQ 15, at 52 weeks. We did not test whether the effect was significant post treatment as this was not a planned trial outcome. In addition to functioning, we assessed improvement using a global change scale, the CGI. An additional response ‘total recovery’ was included on the questionnaire based on the views of a patient representative. Although, no one reported total recovery, patients in the intervention arm were statistically more likely to report feeling better compared to SMC. Interestingly therapists reported that a small number of patients had indeed recovered. This discrepant finding requires further exploration.

Despite PRINCE Secondary showing significant differences in three out of eight secondary outcomes, the patterns of change for all secondary trial outcomes were consistently in favour of TDT-CBT. This suggests that our study intervention had some effect. However, the approach may need to be further developed or changed in terms of intensity to bring about additional meaningful change in outcomes.

PRINCE Secondary included eight sessions of CBT and so future research should assess if more sessions are required or whether booster sessions would be beneficial. Booster sessions have the potential to strengthen behaviour changes by reinforcing the strategies that are presented to patients during the active treatment phase while not introducing any additional material (Schlup, Munsch, Meyer, Margraf, & Wilhelm, 2009). Anecdotal evidence suggests that many of the participants in the trial reported traumatic events in their life which profoundly affected their quality of life. The study took place in London where there are large disparities in social class (Leeser, 2019). This may have influenced the referral to secondary care by the general practitioner. Future studies should ensure there is adequate time to address these complexities as part of the package of care.

Given the approach taken in this study was transdiagnostic it is possible that insufficient tailoring of therapy took place. However, the approach was individualised, and formulation based making this unlikely. Alternatively, it is possible that targeted treatments work better with people who fulfil criteria for specific syndromes. We previously found better effect sizes for IBS specific CBT with the same number of sessions (Everitt et al., 2015, 2019) and better effect sizes are generally found in the trials of CBT for CFS (Malouff, Thorsteinsson, Rooke, Bhullar, & Schutte, 2008; Price, Mitchell, Tidy, & Hunot, 2008). However, although considering the issue of targeted *v.* transdiagnostic approaches one has to consider the issue of complexity. Patients with overlapping symptoms and more than one syndrome may well be more difficult to treat.

A recent study by Schröder et al. (2012) investigated whether group CBT could alleviate symptoms with people who exhibited a range of functional somatic syndromes. The study found that group CBT was more efficacious than enhanced usual care up to 16 months. In comparison with our study, this intervention included four times the number of therapy sessions which suggests that more sessions may have been required in our study, for sustained improvements. However, the primary outcome in Schroder's study was an aggregated score making it impossible to ascertain whether the impact of the intervention was on symptoms or functioning (Schröder et al., 2012).

Almost half of our study population met the criteria for fibromyalgia. A Cochrane review assessed the advantages and harms of CBT for treating fibromyalgia and found that CBT had a small effect on reducing disability [standard mean difference −0.30 (95% CI −0.51 to −0.08)] and negative mood at the end of treatment [standard mean difference −0.33 (95% CI −0.49 to −0.17)]. Long-term data at 6 months showed a moderate effect [standard mean difference −0.52 (95% CI −0.86 to −0.18)] on reducing disability. Our study found a small to medium effect on disability at 20 weeks, suggesting that the transdiagnostic approach was probably more efficacious than targeted treatment for this group (Bernardy, Klose, Busch, Choy, & Häuser, 2013).

The prevalence of MUS is common in females (Jones & de C. Williams, 2019; Poloni et al., 2018). Even so, the percentage of females recruited in our study was higher than expected (86%) with previous recruitment rates ranging from 55% to 76% (Jones & de C. Williams, 2019; Nimnuan et al., 2001a). Almost half of our patients were recruited from rheumatology and therefore it is not surprising that 54% of participants met the criteria for fibromyalgia. This may also explain the large proportion of participants reporting a previous physiotherapy intervention, an evidence-based treatment for pain. However, despite many patients having been offered physiotherapy as a first-line treatment, the evidence suggests that education and CBT as well as exercise are the best non-pharmacological therapies for treating fibromyalgia (Clauw, 2014).

PRINCE Secondary attempted to address many of the methodical issues raised in previous reviews (Liu et al., 2019; Van Dessel et al., 2014). This trial was a well powered study with a long-term follow-up. We recruited patients who described a range of symptoms. Follow-up rates were good reaching 74% at 52 weeks. The therapy was manualised, three therapists were successfully trained, and treatment fidelity outcomes were good. Based on the data we are confident that the intervention is safe as there was no difference between transdiagnostic CBT plus SMC and SMC groups in adverse events or serious adverse events throughout the trial period.

In terms of limitations, however, the generalisability of this trial may have been limited as it was conducted at a single centre and delivered by only three therapists. This trial was originally designed to be a more generalisable study based on the assumption that a representative sample of therapists were going to deliver the therapy. However, prior to the study starting it became clear that it was only feasible for three therapists to be involved. This led to the efficacy of the intervention being evaluated rather than effectiveness in the real world (Chalder et al., 2019). Future trials should consider broadening the geographic area and should include more therapists to carry out a more pragmatic evaluation of the effectiveness of the intervention. Future publications are planned on the cost effectiveness, an investigation of mediators of treatment effects as well as a nested qualitative study exploring the role of culture on the experience and perception of healthcare and daily life in patients with PPS.

## Conclusion

This large RCT evaluated a therapist-delivered transdiagnostic CBT approach because it could potentially treat a range of patients with different PPS as they share symptoms and common cognitive and behavioural responses to symptoms. Our transdiagnostic model and treatment of PPS was not superior to treatment as usual at the final follow-up (52 weeks). Nevertheless, transdiagnostic CBT was associated with improvements in other secondary clinical outcome measures including symptom severity and global improvement. Our intervention also showed an advantage over SMC in changing WSAS at 20 weeks, which was when the active treatment ended. This study needs to be further developed and assessed in a multi-centre study with a larger group of therapists to assess its generalisability.

## Data Availability

No consent was provided for sharing data with third parties. Once papers have been published data will be anonymised and deposited in a repository. Bona-fide researchers can apply to use the data but are required to clearly specify the research question *a priori*.

## Supplementary material

For supplementary material accompanying this paper visit https://doi.org/10.1017/S0033291721001793. click here to view supplementary material

## Study team

Trial managers: Alisia Carnemolla, Sorina Armeanca, Elyse Couch and Meenal Patel. Research workers: Katie Watts, Nicola Ferreira, Jennifer Robertson, Shinal Patel, Paige Fisher-Smith, Abigale Childs and Richard Turner. CBT therapists: David McCormack, Claire Willis and Fabio Simiao. Recruiting consultants: Dr Tom Britton, Dr Biba Stanton, Dr Nora Ng, Dr Toby Garrood, Professor Andy Cope, Dr Sujith Subesinghe, Dr Sardar Bahadur, Professor Ingvar Bjarnason, Dr Guy Chung-Faye, Dr Sukh Chatu, Dr Kumar Pathmanathan, Professor Bruce Kirkham, Dr Alexandra Kent, Dr Surrinder Birring, Dr Geoffrey Warwick, Dr Giovanni Sanna, Dr Luay Zebouni, Dr Dobrina Hull, Dr Catherine Hughes, Dr Patrick Dubois, Dr Bu Hayee, Dr Iona Coltart, and Dr Richard Turner. Administrators: Andrea Bardsley-Ball and Morufat Raji. We would also like to acknowledge all other NHS staff involved in the recruitment of patients to PRINCE Secondary.
