# Supplementary material for: Efficacy of therapist-delivered transdiagnostic CBT for patients with persistent physical symptoms in secondary care: a randomised controlled trial
Source: Psychol Med. 2021 May 31;53(2):486–96. doi: 10.1017/S0033291721001793 (PMC9899570; doi:10.1017/S0033291721001793)
Supplement: Supplementary file 1 [file S0033291721001793sup001.docx]

**Supplementary Table A: Complete case analysis**

|  | N | Estimated treatment effect | (95% CI) | P-value |
| --- | --- | --- | --- | --- |
| Primary outcome |  |  |  |  |
| WSAS 52 weeks | 239 | -0.94 | (-2.94, 1.05) | p=0.352 |
| Secondary outcomes |  |  |  |  |
| WSAS 9 weeks | 264 | -0.06 | (-1.50, 1.13) | p=0.780 |
| WSAS 20 weeks | 259 | -1.50 | (-3.20, 0.20) | P=0.084 |
| WSAS 40 weeks | 243 | -1.82 | (-3.70, 0.06) | p=0.058 |
| PHQ-15 | 237 | -1.60 | (-2.70, -0.49) | p=0.005 |
| PHQ-9 | 238 | -1.42 | (-2.83, -0.01) | P=0.049 |
| GAD-7 | 237 | -1.04 | (-2.29, 0.22) | P=0.105 |
| PPSQ | 227 | -0.37 | (-0.80, 0.06) | P=0.089 |
| CGI | 237 | -0.65 | (-1.07, -0.23) | P=0.002 |

WSAS work and social adjustment scale, PHQ-15 patient health questionnaire – 15 item scale, PHQ-9 patient health questionnaire – 9 item scale, GAD-7 generalised anxiety disorder – 7 item scale, PPS Questionnaire persistent physical symptoms questionnaire, CGI clinical global impression scale,

**Supplementary Figure A: Mean secondary outcome scores by trial arm over time**


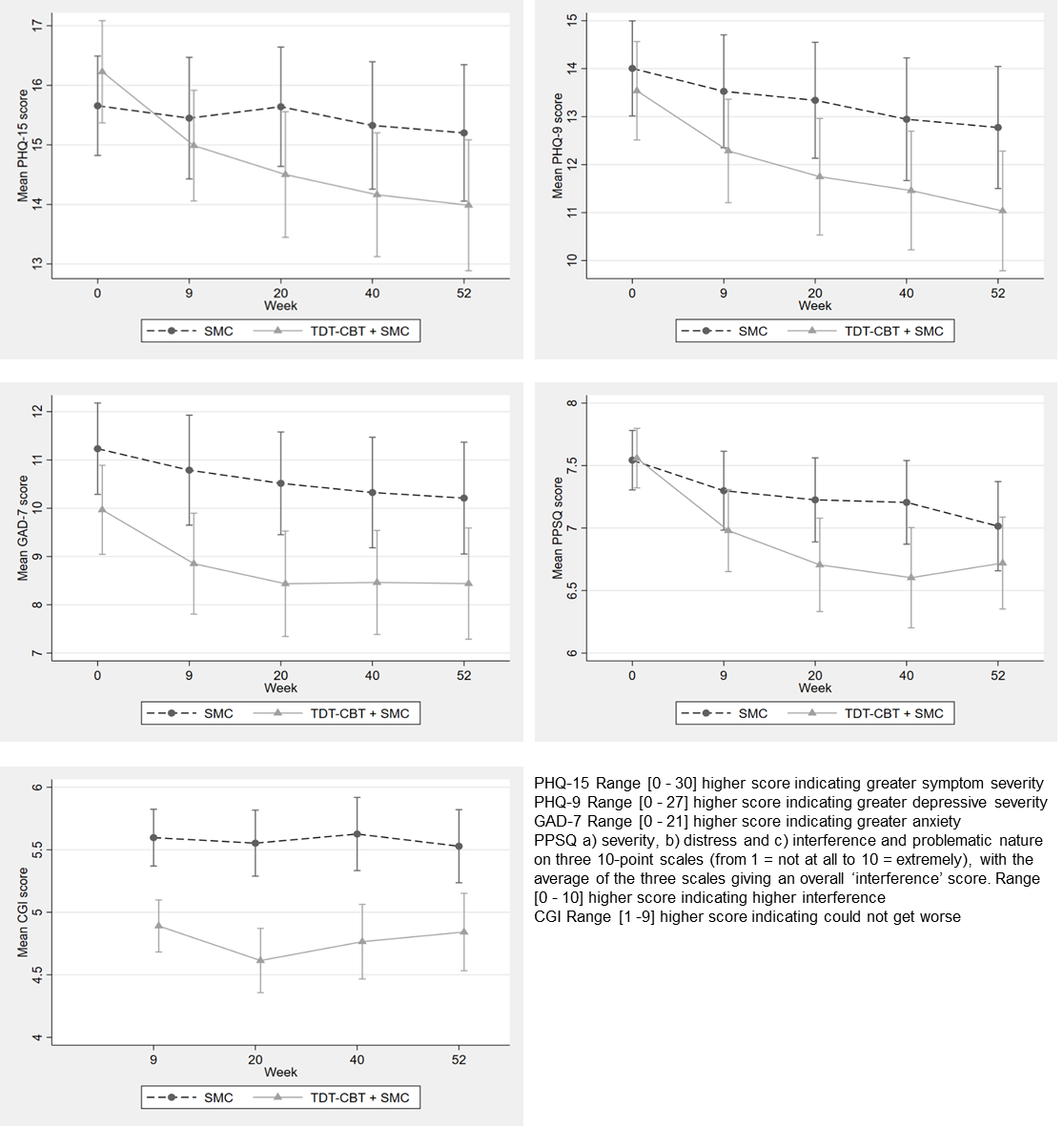


SMC Standard Medical Care: SMC + CBT Therapist delivered, transdiagnostic CBT plus standard medical care; PHQ-15 Patient Health Questionnaire 15, PHQ 9 Patient Health Questionnaire 9, GAD-7 Generalised Anxiety Disorder, PPSQ Persistent Physical Symptom Questionnaire, CGI Clinical Global Impression

**Supplementary Table B: Summaries of outcome measure assessments by trial arm and assessment timepoint**

|  |  | **Baseline** | | | **9** weeks | | | **20 weeks** | | | **40 weeks** | | | **52 weeks** | | |
| --- | --- | --- | --- | --- | --- | --- | --- | --- | --- | --- | --- | --- | --- | --- | --- | --- |
|  |  |  |  |  |  |  |  |  |  |  |  |  |  |  |  |  |
| **Clinical Outcomes** |  | **SMC** | **TDT-CBT plus SMC** | **Overall** | **SMC** | **TDT-CBT plus SMC** | **Overall** | **SMC** | **TDT-CBT plus SMC** | **Overall** | **SMC** | **TDT-CBT plus SMC** | **Overall** | **SMC** | **TDT-CBT plus SMC** | **Overall** |
| **WSAS** | **N** | 163 | 161 | 324 | 134 | 130 | 264 | 132 | 127 | 259 | 123 | 120 | 243 | 124 | 115 | 239 |
| **[0-40]** | **mean (SD)** | 26.6 (8.1) | 26.4 (8.8) | 26.5 (8.4) | 24.7 (9.0) | 23.8 (9.4) | 24.3 (9.2) | 23.8 (10.0) | 21.4 (10.6) | 22.6 (10.3) | 23.9 (10.1) | 21.5 (10.7) | 22.7 (10.5) | 23.0 (10.8) | 21.4 (10.8) | 22.2 (10.8) |
| **PHQ-15** | **N** | 162 | 160 | 322 | 134 | 128 | 262 | 131 | 127 | 258 | 123 | 120 | 243 | 123 | 115 | 238 |
| **[0-30]** | **mean (SD)** | 15.7 (5.4) | 16.2 (5.5) | 15.9 (5.5) | 15.4 (6.0) | 15.0 (5.4) | 15.2 (5.7) | 15.6  (5.8) | 14.5 (6.1) | 15.1 (6.0) | 15.3 (6.0) | 14.2 (5.8) | 14.8 (5.9) | 15.2 (6.5) | 14.0 (6.0) | 14.6 (6.3) |
| **PHQ-9** | **N** | 163 | 161 | 324 | 132 | 129 | 261 | 132 | 127 | 259 | 123 | 120 | 243 | 124 | 114 | 238 |
| **[0-27]** | **mean (SD)** | 14.0 (6.4) | 13.5 (6.6) | 13.8 (6.5) | 13.5 (6.9) | 12.3 (6.2) | 12.9 (6.6) | 13.3  (7.1) | 11.7 (7.0) | 12.6 (7.1) | 12.9 (7.2) | 11.5 (6.9) | 12.2 (7.1) | 12.8 (7.2) | 11.0 (6.8) | 11.9 (7.0) |
| **GAD-7** | **N** | 162 | 161 | 323 | 132 | 128 | 260 | 132 | 127 | 259 | 123 | 119 | 242 | 124 | 114 | 238 |
| **[0-21]** | **mean (SD)** | 11.2 (6.1) | 10.0 (6.0) | 10.6 (6.1) | 10.8 (6.7) | 8.9 (6.0) | 9.8 (6.4) | 10.5  (6.2) | 8.4 (6.3) | 9.5  (6.3) | 10.3 (6.5) | 8.5  (6.0) | 9.4 (6.3) | 10.2 (6.6) | 8.4 (6.3) | 9.4 (6.5) |
| **PPSQ** | **N** | 163 | 161 | 324 | 123 | 124 | 247 | 122 | 124 | 246 | 112 | 114 | 226 | 118 | 109 | 227 |
| **Severity** | **mean (SD)** | 7.1 (1.8) | 7.2 (1.6) | 7.1 (1.7) | 7.0 (1.8) | 6.8 (1.9) | 6.9 (1.8) | 6.9  (2.0) | 6.6 (2.2) | 6.8  (2.1) | 6.9  (1.8) | 6.6  (2.0) | 6.8  (1.9) | 6.8  (1.9) | 6.5  (1.9) | 6.6  (1.9) |
| **Distress** | **mean (SD)** | 7.5 (1.8) | 7.4 (2.0) | 7.4 (1.9) | 7.3 (1.9) | 6.8 (2.1) | 7.1 (2.0) | 7.2  (2.2) | 6.6 (2.4) | 6.9  (2.3) | 7.2  (1.9) | 6.4  (2.5) | 6.8  (2.3) | 6.9  (2.2) | 6.7  (2.3) | 6.8  (2.2) |
| **Interference** | **mean (SD)** | 7.6 (1.9) | 7.6 (1.8) | 7.6 (1.9) | 7.2 (2.2) | 7.0 (2.2) | 7.1 (2.2) | 7.2  (2.3) | 6.7 (2.4) | 6.9  (2.4) | 7.1  (2.3) | 6.6  (2.6) | 6.9  (2.5) | 7.0  (2.4) | 6.8  (2.4) | 6.9  (2.4) |
| **Problematic** | **mean (SD)** | 8.0 (1.7) | 8.0 (1.7) | 8.0 (1.7) | 7.6 (2.1) | 7.3 (2.1) | 7.5 (2.1) | 7.5  (2.1) | 6.9 (2.2) | 7.2  (2.2) | 7.4  (2.0) | 6.8  (2.5) | 7.1  (2.3) | 7.3  (2.4) | 7.0  (2.4) | 7.1  (2.4) |
| **Overall score** | **mean (SD)** | 7.5 (1.5) | 7.6 (1.5) | 7.6 (1.5) | 7.3 (1.8) | 7.0 (1.9) | 7.1 (1.8) | 7.2  (1.9) | 6.7 (2.1) | 7.0  (2.0) | 7.2  (1.8) | 6.6  (2.2) | 6.9  (2.0) | 7.0  (2.0) | 6.7  (2.0) | 6.9  (2.0) |
| **CGI** | **N** | NA | | | 134 | 128 | 262 | 132 | 127 | 259 | 123 | 119 | 242 | 123 | 114 | 237 |
| **[1-9]** | **mean (SD)** | NA | | | 5.6 (1.3) | 4.9 (1.2) | 5.3 (1.3) | 5.6  (1.5) | 4.6 (1.5) | 5.1  (1.6) | 5.6  (1.7) | 4.8  (1.7) | 5.2  (1.7) | 5.5  (1.7) | 4.8  (1.7) | 5.2  (1.7) |

TDT-CBT therapist delivered, transdiagnostic cognitive behavioural therapy intervention, SMC standard medical care, WSAS Work and Social Adjustment Scale, PHQ-15 Patient Health Questionnaire 15, PHQ 9 Patient Health Questionnaire 9, GAD-7 Generalised Anxiety Disorder, PPSQ Persistent Physical Symptoms Questionnaire, CGI Clinical Global Impression
